# Fifth subtype of Kaposi sarcoma in HIV-negative MSM: a retrospective single-arm cohort study from a tertiary care center in NYC from 2000 to 2022

**DOI:** 10.1093/oncolo/oyaf024

**Published:** 2025-03-13

**Authors:** Ayana E Morales, Gabrielle Benson, Stephanie Glavan, Rosemary Giuliano, Mark A Dickson

**Affiliations:** Department of Medicine, Weill Cornell Medicine, New York, NY, United States; Department of Medicine, Memorial Sloan Kettering Cancer Center, New York, NY, United States; Department of Medicine, Memorial Sloan Kettering Cancer Center, New York, NY, United States; Department of Medicine, Memorial Sloan Kettering Cancer Center, New York, NY, United States; Department of Medicine, Weill Cornell Medicine, New York, NY, United States; Department of Medicine, Memorial Sloan Kettering Cancer Center, New York, NY, United States

**Keywords:** Kaposi sarcoma, HIV-negative, men who have sex with men

## Abstract

**Background:**

Kaposi sarcoma (KS) is a vascular tumor caused by human herpesvirus 8, also known as Kaposi sarcoma herpesvirus. There are 4 distinct subtypes: classic, endemic, iatrogenic, and epidemic (HIV-associated). A fifth subtype is increasingly recognized: non-epidemic KS in men who have sex with men (MSM) who are HIV-negative. Our primary objective was to characterize non-epidemic KS to identify associated risk factors, presentation, treatment course, and prognosis of these patients.

**Patients and Methods:**

This retrospective cohort included all patients evaluated at Memorial Sloan Kettering Cancer Center from 2000 to 2022 with pathologically proven KS who identified as MSM status, without diagnosis of HIV. Data were collected on demographics, comorbidities, coinfections, treatments, and outcomes.

**Results:**

Seventy-two patients were identified. The median age at the time of diagnosis was 58. At initial diagnosis, 44% of patients underwent observation, 51% received localized treatment and 5% received systemic treatment. In follow-up, 47% of patients had a progression of disease requiring recurrent treatment: 25% received localized treatment while 18% received chemotherapy. In follow-up, 7 patients died, with only 2 deaths attributed to KS; 10% of patients were diagnosed with a lymphoproliferative disorder.

**Conclusions:**

This study is the largest yet to characterize the non-epidemic KS subtype in HIV-negative MSM. These individuals are younger, HIV-negative, MSM with a favorable prognosis. Additional research is needed to understand the potential risk associated with lymphoproliferative disorders.

**Implications for practice**

It is important to recognize this “Fifth subtype” of Kaposi Sarcoma to identify individuals who are at increased risk for KS despite not having HIV. These results may allow for improved diagnosis and treatment, as well as raising awareness of its overall more favorable prognosis.

## Introduction

Kaposi sarcoma (KS) is an angioproliferative tumor caused by human herpes virus-8 (HHV-8), also known as Kaposi sarcoma herpes virus (KSHV), that occurs in 4 distinct recognized epidemiologic subtypes: classic, endemic, iatrogenic, and epidemic.^1,2^ The histology is similar among the different subtypes, however the presentation and prognosis can vary markedly. The disease develops in the setting of both impaired host immunity and the presence of KSHV infection.^2^ In 1872, Moritz Kaposi first described the classic subtype as an indolent disease that occurred usually in elderly men of Mediterranean or Eastern European Jewish ancestry which typically presents as lesions on the lower extremities in the setting of immunosenescence. This form of KS can be associated with a decreased lymphocyte count.^3^ The endemic subtype occurs in Africa in younger HIV-negative individuals, more common in men than women^4,5^ and may be more aggressive with increased morbidity and mortality.^6^ A lymphadenopathic subvariant of the endemic form in young people, usually without skin lesions, in the setting of chronic concomitant parasitic infections and malnutrition can become rapidly life-threatening.^7,8^ The iatrogenic subtype is related to immunosuppression due to solid organ transplants, other malignancies undergoing immunosuppressive treatment, or auto-immune and rheumatologic conditions. The epidemic form, associated with immunosuppression related to HIV, is usually the most aggressive form seen in people living with HIV in both Western countries and sub-Saharan Africa, and presents with more disseminated disease.^2^ A fifth subtype has recently become increasingly recognized as occurring in HIV-negative MSM (men who have sex with men), without obvious impaired immunity. This fifth subtype was previously described only in case reports and case series, the largest being 28 cases of non-epidemic KS in a multicenter case series in France.^9-15^ It is suggested in the fifth subtype, that a key risk factor is transmission of HHV-8 in high-risk sexual practices.^9,10^ In addition, a meta-analysis of cohort, case-control, and cross-sectional studies revealed a significantly increased odds ratio of having HHV-8 seroprevalence with multiple sexually transmitted infections including gonorrhea, chlamydia, HSV-2, and syphilis.^16^ The nonepidemic KS in HIV-negative men of the fifth subtype usually occurs in MSM of a younger age group compared to the classic subtype who have no identifiable cause for immunodeficiency and have a similar prognosis as individuals with the classic subtype.^14,17^ A recent study conducted in the United States, identified 6 individuals meeting criteria for this subtype confirming the epidemiologic trend amongst a cohort of a total of 72 individuals with other epidemiologic subtypes.^18^ This fifth subtype has not been comprehensively described in nonendemic regions.

Kaposi Sarcoma is definitively diagnosed by biopsy and histopathology, with detection of HHV-8 latency-associated nuclear antigen (LANA) in specimen samples by immunohistochemistry.^2^ Treatment of limited disease, usually in the form of violaceous macules and nodules on the lower extremities, usually consists of observation or localized treatment such as resection, cryotherapy, or topical therapy such as imiquimod,^19^ alitretinoin,^20^ timolol,^21^ or sirolimus.^22^ Patients with HIV may have regression of lesions solely with antiretroviral treatment.^23^ Patients with more advanced disease may require systemic treatment including chemotherapies and immunomodulators that may require repeated cycles and have adverse side effects.^2^ These chemotherapies include pegylated liposomal doxorubicin (PLD)^24,25^ as initial therapy, paclitaxel^26^ in those for whom PLD is contraindicated or as a second line, and an oral option, pomalidomide, an immunomodulatory drug.^27^ HHV-8 is also associated with other diseases that may occur alone or concurrently such as primary effusion lymphoma (PEL), a rare form of non-Hodgkin’s lymphoma; Multicentric Castleman’s disease (MCD), a B-cell lymphoproliferative disorder (LPD); and KSHV inflammatory cytokine syndrome (KICS), which presents in the setting of immunosuppression from HIV as systemic inflammation with increased cytokine levels, however without evidence of KSHV-associated multicentric Castleman disease.^2,28^

The objectives of this study were to characterize the risk factors, clinical presentation, and outcomes of the fifth epidemiologic subtype of KS that were encountered at a major tertiary hospital in New York City, Memorial Sloan Kettering Cancer Center, from 2000 to 2022. We postulated that there is a fifth subtype of KS, occurring in men who have sex with men (MSM), who present at a younger age than classic KS, and who are immunocompetent with a predominantly indolent clinical course and favorable prognosis. We anticipated that these patients are largely from nonendemic countries and may be predominantly managed with active surveillance or local therapy. Additionally, the transgender population, regardless of HIV status may be in particular a disproportionately higher risk for KS, as a study found KS to have the highest proportional incidence ratio (71.7) in a study examining transgender patients diagnosed between 1979 and 2016, with or without HIV.^29^ We sought to define this fifth subtype by age at presentation, extent of disease, histopathologic subtypes, comorbidities, clinical outcomes, and treatment approaches. A previous study by Lanternier in France recognized that while there was a good prognosis for these patients, there was an association with increased risk for lymphoproliferative disorders (Multicentric Castleman’s Disease, Burkitt lymphoma, and follicular lymphoma)^15^ which is potentially clinically relevant. We sought to confirm if these characteristics and others were recognized in our patient population.

## Methods

We performed an observational retrospective study of patients with Kaposi sarcoma encountered at Memorial Sloan Kettering Cancer Center (MSKCC) in New York City from 2000 until 2022 who have met the criteria for the fifth epidemiologic subtype. Institutional Review Board (IRB) approval was reviewed and accepted by the Human Research Protection Program (HRPP) Office of MSKCC to perform this study. The data were collected through a review of the electronic health records (EHR) of all patients either diagnosed with or referred for Kaposi sarcoma at MSKCC in NYC from 2000 to 2022. We performed a chart review, collecting data on age at presentation, sex, gender, sexual orientation, race, ethnicity, location of local or disseminated disease including visceral disease, histopathologic descriptions, documented co-infections, in particular other sexually transmitted infections, chronic comorbidities, documentation of HIV negative status, HIV pre-exposure prophylaxis (PrEP) medication, treatment, and outcomes. Patients were eligible for this study if they presented without HIV diagnosis and were identified as MSM. Data on potential additional risk factors of country of origin from endemic regions, use of immunosuppression, or comorbidities contributing to immunosuppression were also collected, since clinical features may overlap with other forms of KS (classic and iatrogenic). Treatments were defined as: resection, topical therapy (sirolimus, imiquimod, alitretinoin), cryotherapy, laser therapy, oral immunomodulators, and chemotherapy. Clinical outcomes, including response, progression-free survival (the period of time a patient had no evidence of worsening of disease), and overall survival were determined at the time of data collection by review of provider comments in clinical notes. Analysis was conducted using GraphPad Prism version 9.

## Results

**Figure 1.**
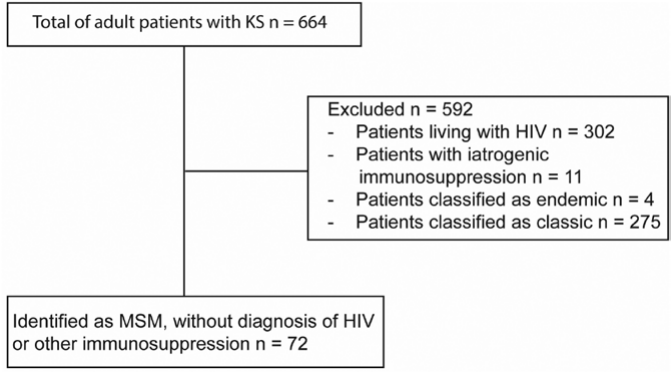
Flowchart of patient inclusion. Abbreviations: HIV, Human immunodeficiency virus; KS, Kaposi sarcoma; MSM, men who have sex with men.

A total of 664 patient charts were reviewed during this study period. The following patients were excluded based on the exclusion criteria: 302 patients living with HIV, 11 patients identified to have a history of iatrogenic KS/solid transplant organ, 4 cases of endemic KS cases, and 275 cases of classic KS. The remaining 72 patients were included in our analysis who identified as MSM and were HIV negative, representing 11% of all patients with KS (Figure 1). Demographic data, co-infections, comorbidities, and clinical characteristics of these individuals are detailed in **Table 1**.

**Table 1.** Clinical characteristics of the HIV-negative non-epidemic KS.

Patient characteristics	*N* = 72	Range
Median age (years)	58.1	32-83
Lower quartile	46.5	
Upper quartile	65.8	
Median duration of follow up (years)	1.95	0-17
Ethnicity/race		
Hispanic/Latino	8 (11%)	
African American or Black	4 (5.5%)	
American Indian/Alaska Native	1 (1.4%)	
Asian	6 (8.3%)	
White American	37 (51.4%)	
White Mediterranean	6 (8.3%)	
White European	10 (13.9%)	
Sexual orientation		
Homosexual	60 (83.3%)	
Bisexual	12 (16.6%)	
HIV PrEP (previous or current)	6 (8.3%)	
CD4 + count (cells/μL) available at KS diagnosis (range = 102-980)	*N* = 13	
<200	1	
200-400	2	
400-1000	10	
>1000	0	
Absolute lymphocyte count (K/μL)	*N* = 29	
< or =0.5	2	
0.5-1	2	
>1-2	12	
>2-3	10	
>3-4	1	
>4	2	
Patient’s initial visit is for recurrent KS	16 (22%)	
*N* = 72		
Number of lesions at diagnosis		
1	40 (55.5%)	
2-5	17 (23.6%)	
>5-10	5 (6.9%)	
>10	3 (4.2%)	
Unknown	7 (9.7%)	
Number of lesions at 1st cancer center visit		
0	11 (15.2%)	
1	19 (26.4%)	
2-5	24 (33.3%)	
>5-10	6 (8.3%)	
>10	10 (13.8%)	
Unknown	2 (2.7%)	
Characteristics of KS lesions		
Upper extremities	15 (20.8%)	
Lower extremities	53 (73.6%)	
Oral mucosa	1 (1.4%)	
Head and neck	3 (4.2%)	
Trunk/Torso	9 (12.5%)	
Genitals	4 (5.5%)	
Lower limb lymphedema	12 (16.7%)	
Bleeding present	5 (6.9%)	
Ulceration present	7 (9.7%)	
Exophytic	1 (1.4%)	
Visceral disease	1 (1.4%)	
Lymph node involvement	1 (1.4%)	
Patient characteristics	*N* = 72	
** History of smoking, former, or current**	24 (33.3%)	
** KPS/ECOG**		
** (100)/ ECOG 0**	35 (48.6%)	
** (90)/ECOG 0**	20 (27.8%)	
** (80)/ECOG 1**	7 9.7%)	
** (70)/ECOG 1**	0	
** (60)/ECOG 2**	1 (1.4%)	
** Unknown ECOG status**	9(12.5%)	
** Previous treatment prior to initial visit**	25 (34.7%)	
** Observation**	1	
** Local therapy**	25	
** Chemotherapy**	4	
** Past medical history: other malignancies**		
** Other lymphoproliferative disorder**	4 (5.6%)	
** Skin squamous or basal cell carcinoma**	8 (11.1%)	
** Melanoma**	5 (6.9%)	
** Prostate cancer**	3 (4.2%)	
** Bladder cancer**	2 (2.8%)	
** Gastric cancer**	1 (1.4%)	
Patient characteristics	*N* = 72	
**Past medical history**		
** Corticosteroid use**	8 (11.1%)	
** Topical steroid use**	4 (5.5%)	
** Oral steroid use**	4 (5.5%)	
** Other immune modifying medications**	3 (4.2%)	
** Autoimmune disease**	4 (5.5%)	
**History of psoriasis**	3 (4.2 %)	
** Diabetes**	7 (9.7%)	
** Hypertension**	28 (38.9%)	
** Hyperlipidemia**	26 (36.1%)	
** Coronary artery disease**	9 (12.5%)	
** Asthma**	5 (6.9%)	
** Hepatitis A**	1 (1.4%)	
** Hepatitis B**	7 (9.7%)	
** Hepatitis C**	0	
** Chlamydia**	3 (4.2%)	
** Gonorrhea**	2 (2.8%)	
** Syphilis**	2 (2.8%)	
** Human papillomavirus (HPV)**	1 (1.4%)	
** Genital herpes simplex virus (HSV)**	7 (9.7%)	

The median age at time of diagnosis of the “Fifth Subtype” was 58 (range: 32-83). Median follow-up was 2 years (range: 0-17). The majority of patients identified as Caucasian (74%). HIV PrEP was previously or at the time of chart review actively being taken by 8.3%. Transgender patients were not identified in this study. One-third of these individuals had a history of smoking, including a current or former history. The majority of patients had good functional performance status determined through provider evaluation of the Karnofsky Performance Status (KPS) and Eastern Cooperative Oncology Group (ECOG) scales, with only minor symptoms and signs of disease.

CD4 lab results were available for only 12 patients, and 3/12 had CD4 < 400. The clinical significance of this in the absence of HIV infection or other identified causes for immunosuppression is not clear. In addition, absolute lymphocyte counts (ALC) at the time of KS diagnosis were available on 29 of the 72 individuals. The majority of patients had normal ALC > 1. One individual at the time of KS diagnosis had an ALC that was abnormally high in the setting of his underlying CLL diagnosis. Overall, more than half of patients had only 1 lesion at the time of diagnosis, and only 11% had > 5 lesions. At this tertiary care center, 22% were presenting with recurrent KS disease.

74% presented with lesions on the lower extremities, with 17% noted to have lymphedema. 5 patients had evidence of bleeding on the exam (3 from the lower extremities, 1 from the neck, and 1 from a lesion in the rectal mucosa). Only one person was found to have evidence of visceral disease, a 90-year-old man with longstanding KS. This patient presented over 30 years prior, at the age of 59, with lesions on his lower extremities that eventually progressed on pegylated liposomal doxorubicin and required multiple rounds of systemic chemotherapy, with eventual spread to lungs which was fatal.

Approximately 10% of patients had a history of corticosteroid use and 10% had a history of diabetes. Of the seven patients with corticosteroid use, 4 of the 7 had oral corticosteroid use prior to the onset of their KS while the other 3 had topical corticosteroid use. Four patients had a history of autoimmune disease: 3 with psoriasis (previously treated with topical steroids and anti-IL-17 therapies) and one with rheumatoid arthritis (treated with steroids, methotrexate, and sulfasalazine). Other comorbidities that were prevalent included hypertension (39%) and hyperlipidemia (36%). Only 10% had a history of Hepatitis B and 10% had genital HSV infection.

Nineteen of the patients had a history of other malignancy preceding or concurrent to their KS diagnosis, 4 with lymphoproliferative disorders, 8 individuals with squamous or basal cell carcinoma of the skin, 5 with melanoma (2 of whom also had non-melanoma skin cancers), 3 with prostate cancer (one of whom had multiple skin cancers), 2 with bladder cancer including one with concomitant gastric cancer.

Our cohort included 10% (7/72) of patients who had evidence of a lymphoproliferative disorder (**Table 2**), with one case of Multicentric Castleman’s disease that was diagnosed concurrently with KS, while 3 lymphoproliferative disorders preceded the KS diagnosis, and 3 lymphoproliferative disorders that succeeded the KS diagnosis. There were 2 chronic lymphocytic leukemias and 4 different types of T-cell lymphoproliferative disorders. Powles et. al. projected the incidence of KSHV-MCD in HIV-positive individuals to be 4.3 cases per 10 000 person-years and observed increasing incidence despite the accessibility of effective ART for HIV.^30^ KSHV-MCD is more likely to occur in individuals with well-controlled HIV, relatively preserved CD4 + T-cell counts and have KSHV-specific CD8 + T-cells.^31,32^

**Table 2.** Description of patients with non-epidemic KS with a history of lymphoproliferative disorder

Patient	Lymphoproliferative disorder (LPD)	Diagnosis of LPD and KS	Description of symptoms of LPD and KS	Treatments for LPD and KS
1	Multicentric Castleman’s disease	KS diagnosed at age of 75. Concurrently diagnosed with KS	Cervical LN biopsy proven MCD and KS. KS skin lesions subsequently developed 3 months following diagnosis.	Rituximab at time of diagnosis,new KS. cutaneous lesions developed3 months later. Recommended for observation of KS lesions.
2	T-cell lymphoproliferative Hyper-eosinophilia syndrome (HES)	KS diagnosed at age of 59. LPD preceded diagnosis of KS by 10 months	Rashes on legs initially began 10 years prior to KS diagnosis.	Prednisone for initial rash and HES. Alpha interferon at time of KS diagnosis and then lost to follow up.
3	Peripheral T-cell lymphoma	KS diagnosed at age 45. LPD succeeded diagnosis of KS by 22 months	Right lower extremity swelling and KS lesions. Developed lymphopenia, then lymphadenopathy (inguinal and cervical).	Cyclophosphamide, doxorubicin, vincristine, etoposide, and prednisone (CHOEP) at time of LPD diagnosis, followed by autotransplant and remission.
4	Chronic lymphocytic leukemia	KS diagnosed at age of 65. LPD succeeded diagnosis of KS by 17 months	Genital KS lesions developed at time of diagnosis. Leukocytosis occurred 12 months after KS diagnosis.	Radiation to initial KS lesions. Pentostatin, rituximab, and cyclophosphamide for CLL worsened KS. Subsequent KS treated with liposomal doxorubicin, followed by paclitaxel, then had POD of CLL, and died of CLL 3 years following initial LPD diagnosis.
5	Chronic lymphocytic leukemia	KS diagnosed at age of 65. LPD succeeded diagnosis of KS by 11 years	Leukocytosis noted on annual physical exam (LPD) 11 years after initial KS diagnosis. Development of KS lesions on genital, perianal, leg, and hip over the span of 18 years.	Initial observation for CLL. Cryoablation for KS, 14 years after KS diagnosis, complicated by cellulitis and sepsis. Multiple topicals for KS 3 years later (sirolimus, imiquimod), liposomal doxorubicin. Leukoreduction for CLL a year later with rituximab ibrutinib, however, KS lesions progressed, followed by liposomal doxorubicin with subsequent stable disease.
6	Mycosis fungoides/cutaneous T-cell lymphoma (CTCL)	KS diagnosed at age of 56. LPD preceded diagnosis of KS by 5 months	Nodular lesions with ulcerations to head/neck/chest/scrotum biopsied 3 months after with abnormal T cell population. KS lesions developed to chest 6 months after LPD diagnosis.	Initially treated with brentuximab and radiation therapy to skin. Rituximab/brentuximab etoposide/doxorubicin 17 months after initial diagnosis. Disease continued to progress; KS recurrence in setting of therapy for LPD. Patient died from lymphoma/respiratory failure 21 months after initial diagnosis.
7	T cell granular lymphocytic leukemia	KS diagnosed at age of 59. LPD preceded diagnosis of KS by 4 months	Chronic neutropenia initially 11 years prior to LPD diagnosis. KS lesions to left foot 3 months after LPD diagnosis.	Initial observation for LPD. Observation for KS, developed new nodule in 6 months after diagnosis, lost to follow-up.

**Figure 2.**
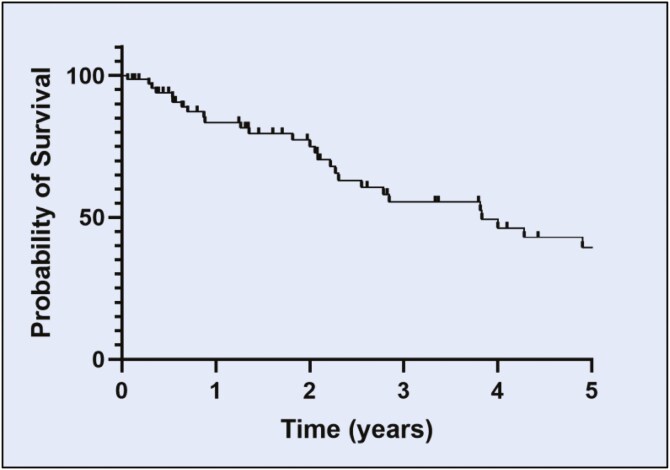
Five year progression-free survival. Five-year progression-free survival of the Fifth subtype of KS of all 72 patients after diagnosis of KS was 39%.

**Figure 3.**
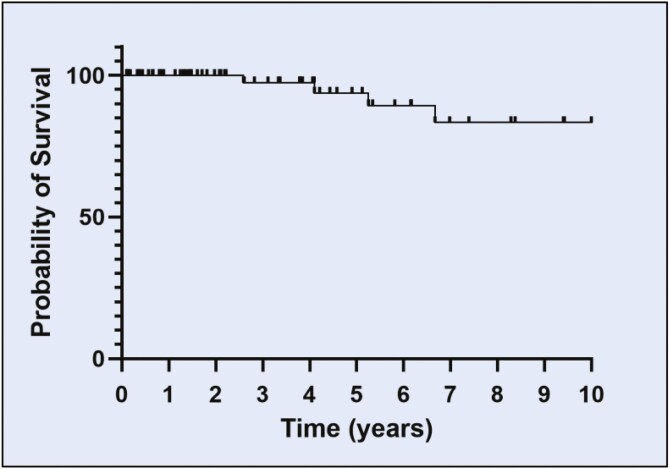
Overall survival. Ten-year overall survival of the Fifth subtype of KS of all 72 patients after diagnosis of KS was 83%.

At initial diagnosis, treatment was observation (active surveillance) for 44% (32/72) of patients, localized therapy for 51% (37/72), and chemotherapy for 5.5% (4/72). 47% (34/72) of patients had progression of disease requiring recurrent treatment. For progressive disease, 25% (18/72) received localized treatment while 18% (13/72) received chemotherapy, including pegylated liposomal doxorubicin and paclitaxel. One unusual case had pathology consistent with KS that evolved into a more aggressive malignancy with some features of angiosarcoma. This patient was treated with systemic therapies used for angiosarcoma (gemcitabine and docetaxel, pazopanib, and sorafenib) and ultimately died of the disease. Systemic chemotherapy was given for a median duration of 14.5 months (range: 3–60 months). Five--year progression-free survival (PFS) was 39% (Figure 2). By the end of the follow-up period, 7 patients had died, with only 2 deaths attributed to KS. 10-year survival for all-cause mortality was 83% (Figure 3), higher than historical data at 65% for all men with KS reported by SEER data.^17^

## Discussion

A new subtype of KS is now increasingly recognized as a non-epidemic variant in a younger population of HIV-negative MSM. This is, to the best of our knowledge, the largest study yet to characterize this disease. The clinical presentation is similar to the classic subtype of KS with a few notable differences. We found in our study that the median age of diagnosis in this fifth subtype was younger than the classic subtype as previously reported.^14^ The median age of diagnosis was 58, younger than the estimated age of onset of classic KS which is estimated at 64-72 years.^2^ Most of these patients had indolent disease and favorable outcomes. Patients from this subtype predominantly presented with disease on the lower extremities with 1/5 of those having lymphedema.

Only 1 patient had evidence of visceral disease for which this individual had long-standing KS that required systemic chemotherapy that unfortunately progressed. Overall 10-year survival of 83%, which is higher than historical data at 65% for all men with Kaposi sarcoma reported by SEER data^17^, and KS was rarely a cause of death. Indeed, most of these patients presented with indolent disease and had favorable outcomes. In our cohort, 10% of patients had a diagnosis of LPD as either a preexisting condition, concurrent with KS diagnosis, or detected during active surveillance. Additional research is needed to understand the potential increased risk of lymphoproliferative disorders.

In identifying patients for this retrospective study, we intentionally cast a wide net, to explore the clinical course of HIV-negative MSM with KS. We acknowledge that the classification of KS into discrete subtypes is imperfect, with potential overlap. For example, a 70-year-old male of Mediterranean ancestry and a diagnosis of rheumatoid arthritis on steroids, whom identifies as MSM has features of classic, iatrogenic, and fifth type KS. Other limitations of this retrospective study include a relatively small sample size due to the rarity of the disease. Potential confounders include referral bias and incomplete information. Some of the patients identified as having fifth type KS had multiple risk factors, including Mediterranean or Eastern European background, and exposure to immunosuppressive treatments, however, these individuals were included in our analysis since their major risk factor for KS may be their MSM status as a risk factor for KSHV transmission. In conclusion, these findings highlight the importance of recognizing this subtype of KS, which can develop in individuals without HIV. These individuals, while they may be similar to classic KS in presentation, may present at a younger age and often have a favorable prognosis. Multicenter studies with a larger sample size would help to confirm these findings.

## Data Availability

The data generated in this study are available upon request from the corresponding author, Mark Dickson (dicksonm@mskcc.org).
